# Anti-*Plasmodium vivax* merozoite surface protein 3 ϒ (PvMSP3 ϒ) antibodies upon natural infection

**DOI:** 10.1038/s41598-024-59153-w

**Published:** 2024-04-26

**Authors:** Napaporn Kuamsab, Chaturong Putaporntip, Azumi Kakino, Rattiporn Kosuwin, Sunisa Songsaigath, Hiroshi Tachibana, Somchai Jongwutiwes

**Affiliations:** 1 Molecular Biology of Malaria and Opportunistic Parasites Research Unit, Department of Parasitology, Faculty of Medicine, Chulalongkorn University, Bangkok, Thailand; 2 Department of Infectious Diseases, Tokai University School of Medicine, Isehara, Kanagawa Japan; 3 Community Public Health Program, Faculty of Health Science and Technology, Southern College of Technology, Nakorn Si Thammarat, Thailand; 4 Department of Health Promotion, Faculty of Physical Therapy, Srinakharinwirot University, Nakhonnayok, Thailand

**Keywords:** Malaria, *Plasmodium vivax*, Merozoite surface protein 3, Anti-PvMSP3 antibody, Coiled-coil structure, Cytophilic antibody, Immunology, Microbiology

## Abstract

Merozoite surface protein 3 of *Plasmodium vivax* (PvMSP3) contains a repertoire of protein members with unique sequence organization. While the biological functions of these proteins await elucidation, PvMSP3 has been suggested to be potential vaccine targets. To date, studies on natural immune responses to this protein family have been confined to two members, PvMSP3α and PvMSP3β. This study analyzed natural IgG antibody responses to PvMSP3γ recombinant proteins derived from two variants: one containing insert blocks (CT1230nF) and the other without insert domain (NR25nF). The former variant was also expressed as two subfragment proteins: one encompassing variable domain I and insert block A (CT1230N) and the other spanning from insert block B to conserved block III (CT1230C). Serum samples were obtained from 246 symptomatic vivax malaria patients in Tak (n = 50) and Ubon Ratchathani (n = 196) Provinces. In total, 176 (71.5%) patients could mount antibodies to at least one recombinant PvMSP3γ antigen. IgG antibodies directed against antigens CT1230nF, CT1230N, CT1230C and NR25nF occurred in 96.6%, 61.4%, 71.6% and 68.2% of samples, respectively, suggesting the widespread occurrence of B-cell epitopes across PvMSP3γ. The rates of seropositivity seemed to correlate with the number of previous malaria episodes. Isotype analysis of anti-PvMSP3γ antibodies has shown predominant cytophilic subclass responses, accounting for 75.4–81.7% for IgG1 and 63.6–77.5% for IgG3. Comparing with previous studies in the same cohort, the numbers of serum samples reactive to antigens derived from *P. vivax* merozoite surface protein 9 (PvMSP9) and thrombospondin-related anonymous protein (PvTRAP) were higher than those to PvMSP3γ, being 92.7% and 87.0% versus 71.5%, respectively. Three (1.22%) serum samples were nonresponsive to all these malarial proteins. Nevertheless, the relevance of naturally acquired antibodies to PvMSP3γ in host protection requires further studies.

## Introduction

*Plasmodium vivax* and *P. falciparum* are the main causative agents of malaria while the former is responsible for relapsing illnesses with wider geographic distribution. Current malaria control strategies targeting malaria parasites, mosquito vectors and people at risk of infections have led to a recent decline in the prevalence of both species in several endemic countries^1^. The biological differences between these *Plasmodium* species seem to be attributed to differential effectiveness of the control measures. During the past decade, an increase in the proportion of vivax malaria has been envisaged amid a decline of the sympatric *P. falciparum* infection in several malaria endemic countries, particularly in Southeast Asian region including Thailand^2^. Therefore, an adjunctive means to control malaria is required such as vaccine development.

Several proteins on the surface coat of malarial merozoites are targets for host immune responses; some of which could elicit interruption of the erythrocyte invasion by the parasites^3^. Merozoite surface protein 3 of *P. falciparum* (PfMSP3) has been considered a promising blood stage vaccine candidate because anti-PfMSP3 antibodies were associated with clinical protection and reduced risk of malaria^4–7^. In *P. vivax*, merozoite surface protein 3 (PvMSP3) comprises a repertoire of antigenically distinct but related proteins encoded by the *Pvmsp3* gene family in which 12 putative members have been identified in the Salvador I strain of *P. vivax*^8^. Despite extensive sequence variation among members in the PvMSP3 multigene family, most of them including PvMSP3α, PvMSP3β and PvMSP3γ possess central complex heptad repeats encoding alanine-rich coiled-coil structure^9–12^. Importantly, almost all *Pvmsp3* gene members seem to be actively expressed during blood stage infections while the timing and magnitude of gene expression could differ during asexual blood stage development^13^. Although *P. vivax* does not possess authentic homolog of PfMSP3, it has been hypothesized that PvMSP3 and PfMSP3 could have undergone convergent evolution in their encoded protein functions for erythrocyte invasion by the merozoites^8^.

Both PvMSP3α and PvMSP3β were immunogenic upon natural infection, characterized by differential IgG reactivity across the protein domains^14–17^. Furthermore, naturally acquired antibodies to PvMSP3α and PvMSP9 were associated with reduced risk of subsequent vivax malaria in Papua New Guinean children^17^. Although naturally acquired antibodies to other protein members in the PvMSP3 family have not been investigated, there remains a possibility that PvMSP3γ could also be a vaccine target.

For PvMSP3γ, our previous sequence analysis of clinical isolates has revealed that variation in this protein can be classified into 3 major types, represented by the Belem, Salvador I and NR520 strains. The Belem type contains insert blocks A, B and C whereas the Salvador I type lacks insert block B. On the other hand, the NR520 type does not possess any insert block^12^. Although imperfect nucleotide repeats have been identified in six regions of the gene, none encoded amino acid repeats. PvMSP3γ contained more than 20% of acidic amino acids similar to those observed in PvMSP3α and PvMSP3β^9,11,12,18,19^. Analysis of the complete PvMSP3γ sequences among clinical isolates in Thailand has shown remarkable population structure of *P. vivax* while differential prevalence of the three major types were found across malaria endemic areas of the country^12^.

To date, studies on natural antibody responses to PvMSP3 have been confined to PvMSP3α and PvMSP3β. In this study, we determined IgG antibody reactivity to two major types and different domains of PvMSP3γ. IgG isotype responses to this protein have been analyzed. Furthermore, we compared anti-PvMSP3γ antibody response profiles with previously analyzed antibody reactivity to *P. vivax* thrombospondin-related anonymous protein (PvTRAP)^20^ and merozoite surface protein 9 (PvMSP9)^21^. Results revealed that during *P. vivax* infection PvMSP3γ elicited IgG antibody responses of predominantly cytophilic subclasses. Despite immunogenicity of PvMSP3γ, the overall seropositivity rate seems to be remarkably lower than PvTRAP and PvMSP9 based on serological analysis of the same cohort.

## Results

### Blood samples

Venous blood samples were obtained from 246 symptomatic vivax malaria patients from Tak (n = 50) and Ubon Ratchathani (n = 196) Provinces. These blood samples were previously analyzed for IgG antibodies against PvTRAP^20^ and PvMSP9 recombinant proteins^21^. The age range of the patients varied from 2 to 65 years with a mean age of 30.9 years. Of these, 204 patients were male (82.9%). All patients had *P. vivax* monoinfections as confirmed by species-specific PCR^22^.

### Expression of recombinant PvMSP3γ proteins

**Figure 1:**
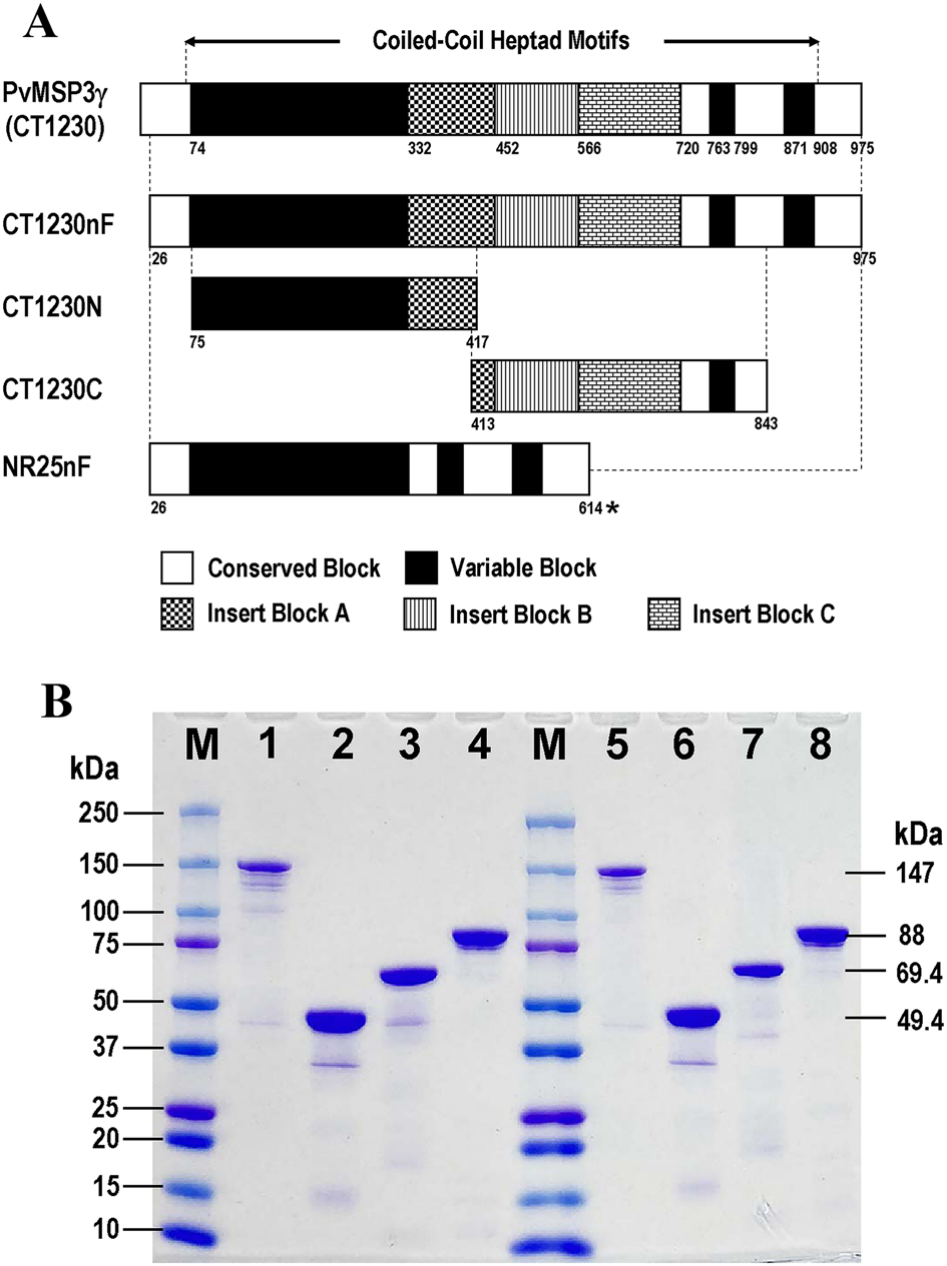
(**A**) Structure of PvMSP3γ showing boundaries of conserved, variable and insert blocks. Approximate locations of recombinant proteins are shown as broken lines corresponding to the positions in the CT1230 sequence with amino acid positions at the N-terminal and C-terminal ends of each protein. Astersk denotes corresponding position 975 of the CT1230 sequence. (**B**) SDS-PAGE of purified PvMSP3γ recombinant proteins stained with Coomassie brilliant blue. Lane M indicates molecular weight marker. Lanes 1–4 in respective order are proteins CT1230nF, CT1230N, CT1230C and NR25nF under reducing condition (with β-mercaptoethanol). Lanes 5–8 are the corresponding proteins under non-reducing condition (without β-mercaptoethanol). See Supplemental Fig. S3 for the uncropped gel image.

Two prevalent *Pvmsp3γ* haplotypes in Thailand, represented by isolates CT1230 and NR25, were used as templates for the production of antigens^12^. The former belonged to the Belem type containing complete insert blocks A, B and C whereas the latter lacked these insertions (Supplemental Fig. S1). Near full-length proteins excluding the N-terminal signal peptides (amino acid residues 1-25) of both haplotypes were expressed as His-tagged fusion proteins, designated CT1230nF and NR25nF (Fig. 1A). Two additional protein fragments of the CT1230 haplotype were produced, one spanning from variable block I to insert block A (CT1230N) and the other from the C-terminal portion of insert block A to the C-terminal end of the protein (CT1230C) (Fig. 1A and Supplemental Fig. S2). The purified recombinant proteins CT1230nF, CT1230N, CT1230C and NR25nF measured approximately 147, 49.4, 69.4 and 88 kilodaltons (kDa), respectively, on both reducing and non-reducing sodium dodecyl sulfate polyacrylamide gel electrophoresis (SDS-PAGE) (Fig. 1B and Supplemental Fig. S3). The molecular weights of these proteins exceeded their respective predicted masses based on their amino acid compositions, being 105.4, 39.5, 48.8 and 66.8 kDa with the estimated isoelectric point (pI) values of 5.11, 6.00, 6.64 and 5.36, respectively. After adjusting for the composition of acidic amino acids^23^, the respective predicted molecular weights of these proteins yielded 134.6, 48.2, 59.2 and 82.7 kDa which were close to the observed migration of the proteins in SDS-PAGE.

### Prevalence of IgG antibodies to the recombinant PvMSP3γ antigens

In total, 176 of 246 (71.54%) patients could mount IgG antibodies to at least one of the PvMSP3γ antigens. Antigen CT1230nF was most frequently recognized by the IgG antibodies (170 of 246 serum samples, 69.11%). Antibodies to antigens CT1230N, CT1230C and NR25nF could be detected in 43.90%, 51.22% and 48.78% of all samples, respectively (Table 1). The percentage of responders to antigen CT1230nF was significantly greater than those to other antigens (χ^2^ test, *p* < 10^–4^). The prevalence of anti-PvMSP3γ antibodies did not significantly differ between patients from Tak and Ubon Ratchathani Provinces (χ^2^ test, *p* > 0.05). Likewise, the percentages of responders and antibody reactivity indices to each antigen were not remarkably different between genders (Fisher’s exact test, *p* > 0.05) and age groups of the patients (paired *t* test, *p* > 0.05) (Supplemental Tables S1–S3).

**Table 1:** Prevalence of IgG antibodies to PvMSP3γ among 246 vivax malaria patients.

	Total IgG antibodies to antigen				
	CT1230nF	CT1230N	CT1230C	NR25nF	Total #
Ranges of OD_490_ values	0.002–3.079	0.002–2.973	0.003–3.004	0.002–3.010	–
Mean OD_490_ ± S.D.	0.922 ± 0.834	0.389 ± 0.551	0.884 ± 0.834	0.520 ± 0.674	–
Median OD_490_	0.705	0.199	0.475	0.237	–
IQR OD_490_ values	0.223, 1.296	0.065, 0.462	0.065, 0.462	0.099, 0.628	–
Cut-off OD_490_ values	0.432	0.237	0.446	0.256	–
Distribution of responders					
Tak (n = 50)					
Seropositive (%)	31 (62)	25 (50)	22 (44)	20 (40)	32 (64.00)
Seronegative (%)	19 (38)	25 (50)	28 (56)	30 (60)	18 (36.00)
Ubon Ratchathani (n = 196)					
Seropositive (%)	139 (70.92)	83 (42.35)	104 (53.06)	100 (51.02)	144 (73.47)
Seronegative (%)	57 (29.08)	113 (57.65)	92 (46.94)	96 (48.98)	52 (26.53)
All (n = 246)					
Seropositive (%)	170 (69.11)	108 (43.90)	126 (51.22)	120 (48.78)	176 (71.54)
Seronegative (%)	76 (30.89)	138 (56.10)	120 (48.78)	126 (51.22)	70 (28. 46)

^#^Positives for at least one antigen.

### Patterns of antibody responses to PvMSP3γ antigens

**Figure 2:**
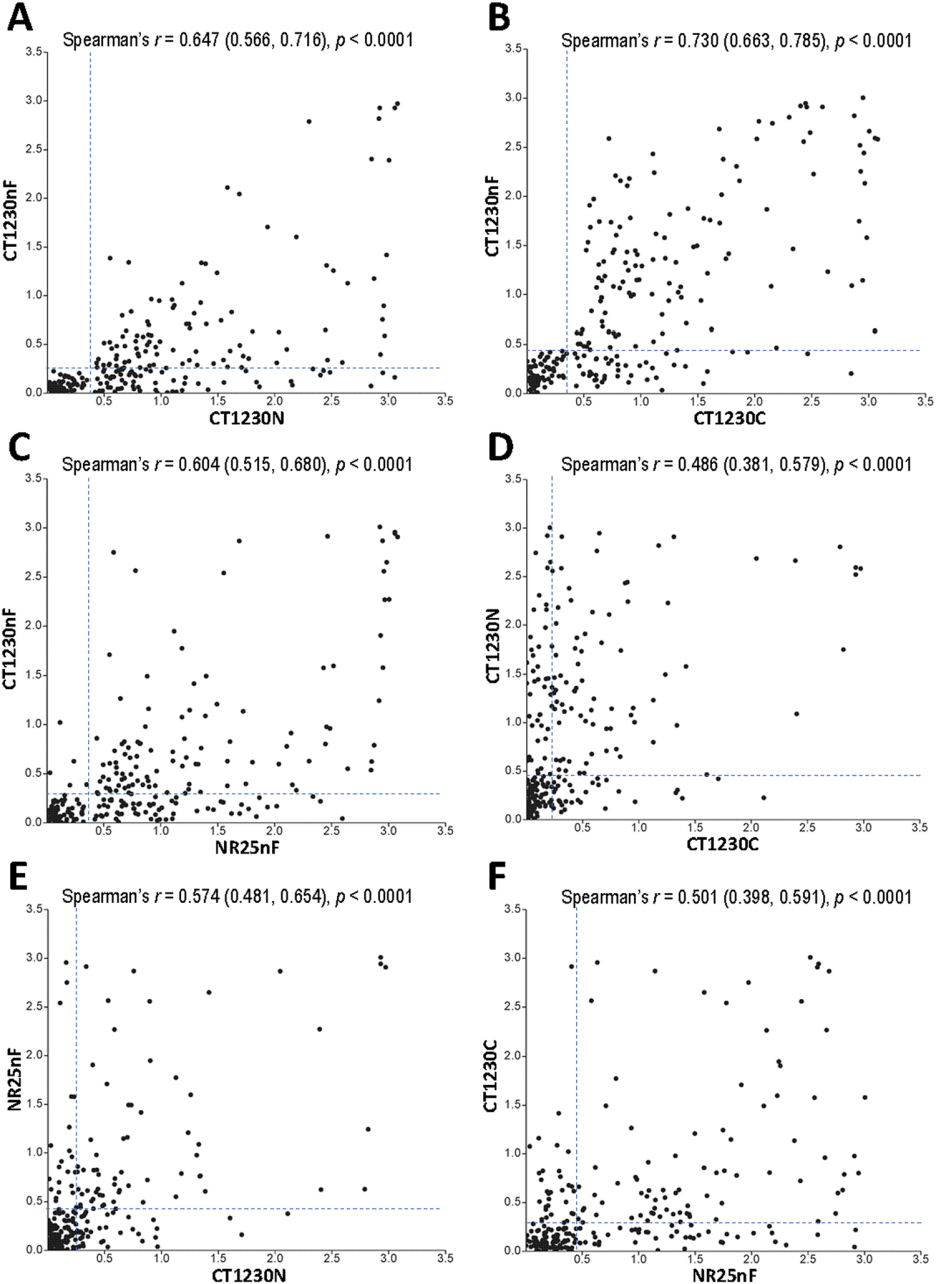
Scatterplot of serum IgG antibody reactivity (OD_490_ values) to antigens CT1230nF, CT1230N, CT1230C and NR25nF in pairwise comparisons (**A–F**). Horizontal and vertical broken lines indicate cutoff values for respective antigens.

Of 170 seropositive samples to antigen CT1230nF, 108 and 126 samples possessed IgG antibodies that recognized antigens CT1230N and CT1230C (Table 2). None of the seronegative samples to antigen CT1230nF was reactive to either antigen CT1230N or CT1230C. Exclusive response to either antigen CT1230N or CT1230C occurred in 31 and 49 samples, respectively, accounting for 18.23% and 28.82% of CT1230nF-seropositive patients (Supplemental Table S4). Simultaneously positive antibody responses across antigens CT1230nF, CT1230N and CT1230C were found in 77 samples, accounting for 31.3% of total samples or 49.05% of 157 samples reactive to either CT1230N or CT1230C. Meanwhile, 56 of 170 CT1230nF-seropositive serum samples were not reactive to antigen NR25nF and vice versa was observed in 6 samples (Table 2). Although the OD_490_ values of total IgG antibodies to each antigen in all pairwise comparisons yielded a significant correlation (Permutation test, *p* < 0.0001), the magnitude of Spearman’s correlation coefficient (*r*) values varied from 0.486 to 0.730 (Fig. 2A–F). Importantly, a two-tailed 95% confidence interval of the *r* value for reactivity to antigens CT1230N and CT1230C was between 0.381 and 0.579 while it was between 0.398 and 0.591 for antigens NR25nF and CT1230C (Fig. 2D and F). Based on a conventional approach to interpreting a correlation coefficient, they were relatively low and could be arbitrarily considered as weak correlation or unimportant relationship and marginal correlation (*r* between 0.10 and 0.39 for weak correlation)^24^. A number of serum samples with the OD_490_ values above the cutoff level for antigen CT1230N did not have positive antibody levels to antigen CT1230C and vice versa (Fig. 2D). A similar contradictory profile of the OD_490_ values was observed for antibody reactivity to antigens CT1230C and NR25nF (Fig. 2F).

**Table 2:** Profile of IgG antibodies to recombinant PvMSP3γ antigens relative to seropositivity to antigen CT1230nF.

	No. serum sample reactive to antigen (% of 246 samples)						
	CT1230N		CT1230C		NR25nF		Total
	Positive	Negative	Positive	Negative	Positive	Negative	
CT1230nF seropositive	108 (43.90)	62 (25.20)	126 (51.22)	44 (17.89)	114 (46.34)	56 (22.76)	170 (69.11)
CT1230nF seronegative	0 (0)	76 (30.90)	0 (0)	76 (30.89)	6 (2.44)	70 (28.46)	76 (31.09)
Total	108 (43.90)	138 (56.10)	126 (51.22)	120 (48.98)	120 (48.78)	126 (51.22)	246 (100)

### Previous malaria exposure and anti-PvMSP3γ antibodies

The relationship between antibody reactivity to each antigen and the number of previous malaria exposure was analyzed from 174 patients who could provide self-report information and/or diagnosis made by malaria clinics or local district hospitals^21^. Of these, 53 and 30 patients had single and multiple (more than once) malaria exposures, respectively. The remaining 91 patients were malaria-naïve prior to the current infection. Results revealed that the prevalence of seropositive antibodies to each antigen seemed to correlate with previous malaria episodes (χ^2^ for trend, *p* < 0.05) (Table 3). Likewise, the levels of reactivity indices of antibody responses to each antigen were positively associated with the number of previous malaria attacks (Kruskal–Wallis *H* test, *p* < 0.05) (Table 4).

**Table 3:** Previous malaria exposure and prevalence of seropositivity to PvMSP3γ antigens.

No. previous malaria episodes	n	No. positives antibodies to antigens (% responders)				
		CT1230nF	C1230N	CT1230C	NR25nF	Total#
0	91	55 (60.44)	36 (39.56)	39 (42.86)	35 (38.46)	55 (60.44)
1*	53	45 (84.91)	27 (50.94)	38 (71.70)	35 (66.04)	46 (86.79)
2–4**	30	26 (86.67)	18 (60.00)	21 (70.00)	19 (63.33)	27 (90.00)
Chi-square for trend		11.57	4.36	10.92	9.28	14.70
*p* value		0.0007	0.0368	0.0010	0.0023	0.0001

^#^Positives for at least one antigen.

*Time to last exposure: range 2–7 years (mean ± S.D. = 3.47 ± 1.41 years) based on 30 patients.

**Time to last exposure: range 2–8 years (mean ± S.D. = 3.98 ± 1.60 years).

**Table 4:** Reactivity indices and OD_490_ values of anti-PvMSP3γ antibodies relative to previous malaria exposure.

No. previous malaria episodes	n	Mean OD_490_ ± S.E. to antigens (Mean reactivity indices ± S.E.)			
		CT1230F	CT1230BI	CT1230BII	NR25F
0	91	0.811 ± 0.086 (1.881 ± 0.200)	0.367 ± 0.059 (1.554 ± 0.252)	0.804 ± 0.091 (1.807 ± 0.204)	0.467 ± 0.071 (1.832 ± 0.277)
1	53	1.179 ± 0.115 (2.734 ± 0.268)	0.465 ± 0.079 (1.972 ± 0.335)	1.191 ± 0.112 (2.676 ± 0.253)	0.758 ± 0.109 (2.973 ± 0.428)
2–4	30	1.154 ± 0.161 (2.678 ± 0.373)	0.559 ± 0.124 (2.367 ± 0.527)	0.968 ± 0.138 (2.175 ± 0.310)	0.655 ± 0.145 (2.569 ± 0.568)
Cut-off value		0.432	0.237	0.446	0.256
Kruskal–Wallis *H*		11.530	9.695	11.660	12.080
*p* value		0.003	0.008	0.003	0.002

### IgG subclass responses

**Figure 3:**
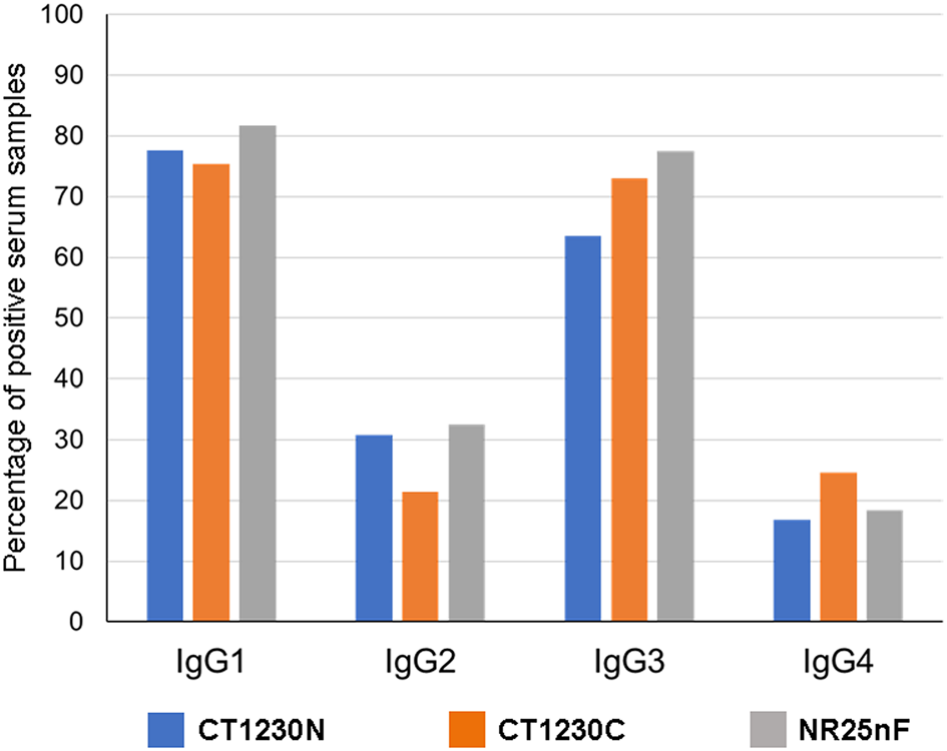
Percentages of IgG subclass responses to recombinant PvMSP3γ antigens.

**Figure 4:**
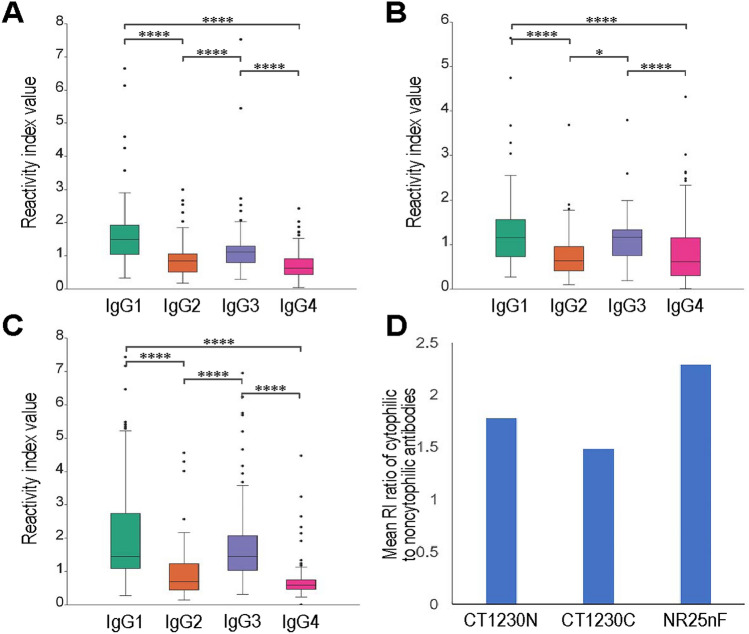
Reactivity index (RI) values of IgG subclass responses to antigens CT1230N (**A**), CT1230C (**B**) and NR25nF (**C**) and mean reactivity index (RI) ratio of cytophilic to noncytophilic antibodies for each antigen (**D**). Median RI value is shown as a horizontal line inside each box plot (**A–C**). * *p* < 0.05 and **** *p* < 0.0001 (Mann–Whitney *U* test).

IgG subclasses were measured from seropositive samples to antigens CT1230N (n = 108), CT1230C (n = 126) and NR25nF (n = 120). Most IgG antibodies to these antigens belonged to IgG1 and IgG3 subclasses (Fig. 3). A slight difference in the positive rates was observed for IgG1 antibodies to antigens CT1230N, CT1230C and NR25nF, accounting for 77.57%, 75.40% and 81.67% of their respective seropositive samples. Meanwhile, 63.55%, 73.02% and 77.50% of seropositive samples for antigens CT1230N, CT1230C and NR25nF, respectively, contained IgG3 subclass. Both IgG2 and IgG4 subclass responses to these antigens were observed in a smaller number of samples, ranging from 21.43 to 32.50% for IgG2 and from 16.82 to 24.6% for IgG4 (Fig. 3 and Supplemental Table S5). The mean reactivity index values of IgG1 and IgG3 antibodies were significantly greater than those of IgG2 and IgG4 subclasses (*t*-test, *p* < 0.05) (Fig. 4A–C). The overall ratios of reactivity index values of cytophilic (IgG1 and IgG3) to noncytophilic (IgG2 and IgG4) antibodies for antigens CT1230N, CT1230C and NR25nF were 1.78, 1.48 and 2.25, respectively (Fig. 4D). The effect of previous malaria exposure on the association trend for the level of reactivity index values of IgG subclasses could not be computed due to inadequate sample size of some categorical data.

### Comparative antibody responses to PvMSP3γ, PvMSP9 and PvTRAP

**Figure 5:**
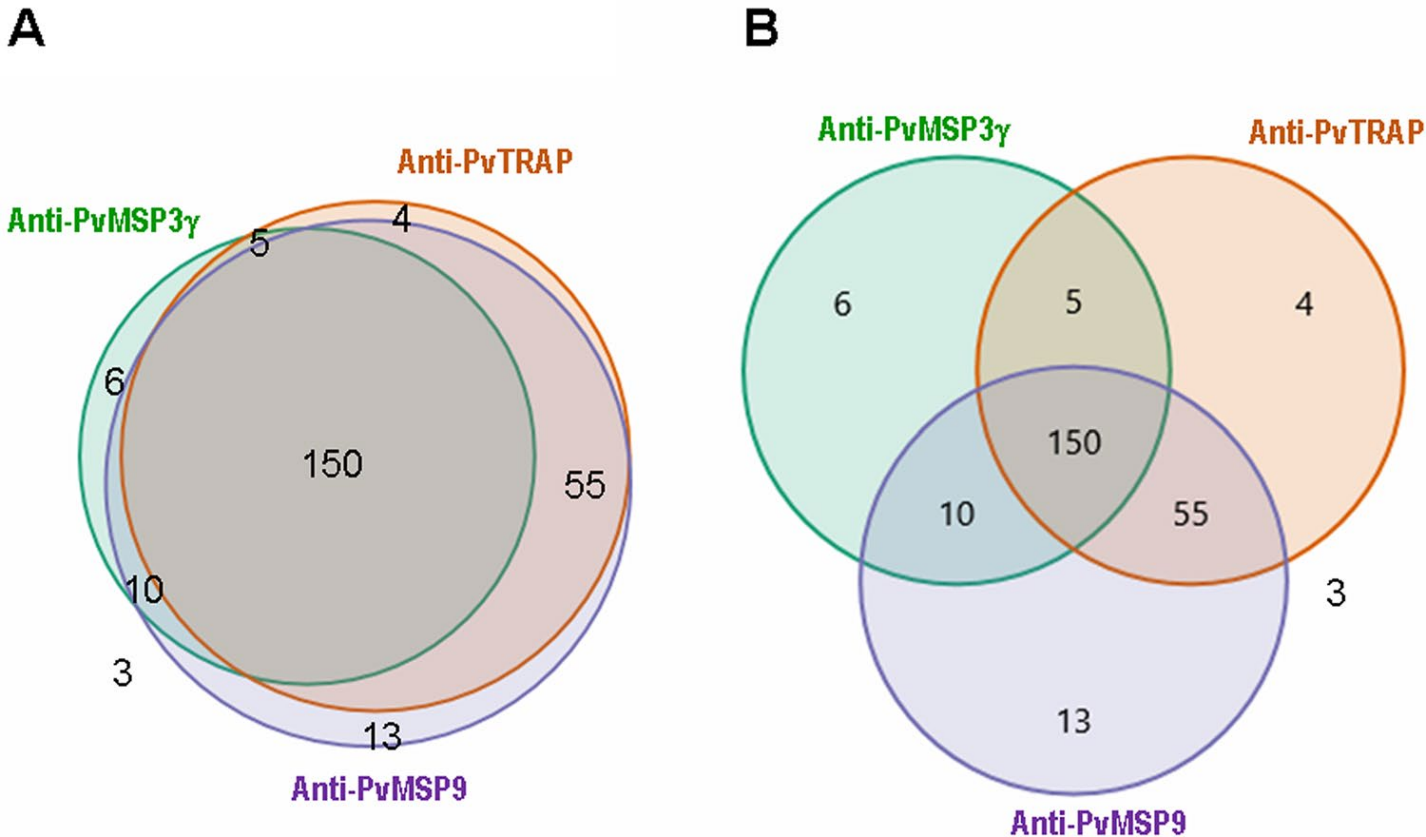
Proportional (**A**) and nonproportional (**B**) plots of Venn diagram representing the number of isolates having antibody reactivity to recombinant PvMSP3γ, PvMSP9 and PvTRAP antigens. Three isolates outside all circles denote no antibody reactivity to all antigens.

To investigate humoral immune responses to different malarial proteins, we compared IgG antibody response to PvMSP3γ with those directed against PvTRAP and PvMSP9 from our previous analysis using serum samples from the same patients^20,21^. Of 246 serum samples, 214 (86.99%) samples had IgG antibodies to at least one of eight recombinant antigens representing 4 variants each for domains II and IV of PvTRAP^20^. For PvMSP9, 228 (92.68%) patients could mount antibodies to at least one of four recombinant antigens derived from two variants each for the N- and C-terminal domains of the protein^21^. Although the rates of seropositivity to PvTRAP and PvMSP9 were not significant differencee (χ^2^ test, *p* > 0.05), they were greater than the seropositive rate of PvMSP3γ (71.55%) (χ^2^ test, *p* < 0.05). In total, 150 (60.98%) patients had antibodies to all 3 malarial proteins (Fig. 5) whereas only 3 (1.22%) serum samples were not responsive to any of these antigens. Of 174 patients with known history of malaria exposure, there was no significant association between the rates of seropositivity to one and more than one of these proteins and history of previous malaria exposure (χ^2^ test, *p* > 0.05) (Supplemental Table S6).

## Discussion

Like PvMSP3α and PvMSP3β, PvMSP3γ was capable of inducing IgG antibody response upon *P. vivax* infection. Previous seroepidemiological studies have shown that approximately 68% and 78% of people living in malaria endemic areas of Brazilian Amazon had IgG antibody to recombinant antigens derived from the central and near full-length sequences of PvMSP3α, respectively^14,16^. Consistently, about 79.1% of *P. vivax* infected individuals in Brazilian Amazon developed antibodies to PvMSP3β antigens^16^. The overall prevalence of anti-PvMSP3γ antibodies in this study was 71.59% which was almost comparable with the seropositive rates for PvMSP3α and PvMSP3β, suggesting an overall similar immunogenicity of these PvMSP3 protein members. Although both PvMSP3α and PvMSP3β seemed to be highly immunogenic upon natural malaria exposure, comparative analysis of concurrent serum samples revealed consistently higher frequency of responders (86.7% to ~ 94%) to the C-terminal 19 kDa fragment of *P. vivax* merozoite surface protein 1 (PvMSP1)^14–16^. It is apparent from our studies that the numbers of serum samples reactive to PvTRAP (86.99%) and PvMSP9 (92.68%) antigens were significantly more than that to PvMSP3γ^20,21^. It is noteworthy that the magnitudes of mean amino acid distance (± standard error) in PvTRAP and PvMSP9 were almost comparable (0.00728 ± 0.00187 and 0.00837 ± 0.00169, respectively; z test, *p* = 0.66) while both were statistically lower than that in PvMSP3γ (0.33147 ± 0.01488; z test, *p* < 0.00001)^25,26^. It could be that in areas of low malaria transmission, seroepidemiological studies might reveal a higher frequency of responders to conserved antigens rather than to a representative variant form of polymorphic antigens that may have a tendency to induce allele-specific antibody response^14–16,20,21^. For PvMSP9, allele-specific antibody responses to the N-terminal and C-terminal domains occurred in 15.9% and 16.7% of seropositive individuals, respectively, while anti-PvTRAP antibodies exclusively reactive to particular alleles in domains II and IV of the protein were observed in 26.1% and 9.7% of seropositive subjects^20,21^. Therefore, some representative polymorphic malarial antigens used in seroepidemiological studies could underrate the overall prevalence of antibody responses in the surveyed population. Furthermore, the use of refolded protein in the ELISA may give rise to further underrating of the responses, as the refolded protein may not have adopted the native conformation of the protein completely. Alternatively, host genetic factors could influence the immune responses to malarial proteins. A comprehensive analysis of human leucocyte antigens (HLA) class II alleles among individuals in a Brazilian endemic area has revealed that differential antibody reactivity to PvMSP1, PvMSP3α and PvMSP9 was associated with particular HLA-DRB1 alleles^27^. Taken together, analysis of cross-sectional blood samples could potentially underestimate the true prevalence of antibody reactivity to malarial proteins.

Our previous study has shown differential prevalence of PvMSP3γ haplotypes across major malaria endemic provinces of Thailand^12^. Importantly, isolates bearing the Belem type sequences were most common in Tak and Ubon Ratchthani Provinces while the NR520 type circulated in these provinces with much lower frequencies. In this study, antigen CT1230nF belonging to the Belem type was the most common target for antibody recognition accounting for 69.11% of the total samples while a significantly lower seropositive rate for antibody reactivity to antigen NR25nF (NR520 type) was observed, suggesting that circulating parasite strains could influence antibody reactivity to allelic types of PvMSP3γ. Besides microheterogeneity of sequences in the conserved blocks of antigens CT1230nF and NR25nF, a striking difference between the two antigens was the presence of three insert domains in the former protein. It is noteworthy that only 6 of 120 (5%) NR25nF-seropositive samples were not reactive to antigen CT1230nF whereas 56 of 170 (32.94%) CT1230nF-seropositive samples were not reactive to antigen NR25nF (Table 2). Therefore, it is likely that the higher prevalence of antibody reactivity to antigens CT1230nF than NR25nF could be mainly attributed by the presence of B-cell epitopes in the insert blocks rather than microheterogeneity of epitope sequences in the conserved domains per se. Intriguingly, it seems that the insert blocks in PvMSP3γ may not be essential for the parasite survival because *P. vivax* isolates bearing the NR25 (NR520) type were three times more prevalent than those harboring the CT1230 (Belem) type in southern Thailand^12^. It is worth noting that low complexity regions (LCRs) or segments of a protein (or DNA) which are biased in amino acid (or nucleotide) composition have been predicted to locate interspersedly in PvMSP3α^28^. The PvMSP3γ recombinant proteins in this study exhibited similarly abundant LCRs encompassing mainly variable and insert blocks (Fig. 1A and Supplemental Fig. S4). Low complexity regions in PvMSP3α have been suggested to confer immune evasion through sequence motifs mimicking B-cell epitopes^28^. Furthermore, LCRs have been shown to mediate antigenic diversity conferring immune escape mechanisms of pathogens^28–30^. None of the seronegative samples to antigen CT1230nF was reactive to the cognate subfragments (CT1230N and CT1230C) which was in line with the possible corresponding B-cell epitope coverage (Figs. 1, 2A and B). On the other hand, 13 of 170 (7.64%) CT1230nF-seropositive samples were nonreactive to either of these subfragment antigens, implying the presence of immunogenic B-cell epitopes in the N-terminal and C-terminal ends of the protein. Therefore, B-cell epitopes have a wide distribution across PvMSP3γ.

Exclusive antibody reactivity to antigen CT1230N occurred in 18.23% of 170 CT1230nF-seropositive individuals while 28.82% of these patients had antibodies reactive only to antigen CT1230C (Table 2 and Supplemental Table S4). These antibodies may recognize distinct epitopes in each subfragment antigen. Meanwhile, 77 (45.29%) CT1230nF-seropositive patients could mount antibodies to both subfragments (Table 2). Both antigens CT1230N and CT1230C shared only 5 overlapping amino acids in insert block A (Supplemental Fig. S2). These short overlapping amino acid residues were unlikely responsible for simultaneous reactivity of antibodies to both antigens. On the other hand, it is plausible that a repertoire of antibodies in each serum sample may recognize diverse epitopes located in these antigens. Interestingly, the reactivity index values of serum samples simultaneously reactive to both antigens did not show statistically meaningful correlation (n = 77, Pearson’s *r* = 0.22867, 95% CI [0.0049, 0.43]) because the lower limit of 95% confidence interval falls within an arbitrary level for negligible correlation^24^, suggesting heterogeneous antibody reactivity within most samples.

Meanwhile, the α-helical secondary structure and coiled-coil tertiary structure are ubiquitous in protein sequences that mediate both intra- and intermolecular protein–protein interactions^31^. Besides having important roles in cellular architecture and function^32^, coiled-coil structure of proteins has been implicated in inducing cross-reactive antibodies due to its capability to form similar conformational epitopes between antigens. Therefore, an alternative explanation for simultaneous antibody reactivity to both CT1230N and CT1230C antigens in almost half of the serum samples (77 of 157 samples) reactive to either of these antigens could probably stem from cross-reactive epitopes in these antigens. Although coiled-coil heptad repeat sequence identity could contribute to cross-reactive epitopes, it seems that amino acid identity/similarity at solvent-exposed positions in heptad repeats may influence antibody cross-reactivity, an issue responsible for antibody cross-reactivity among several variants of streptococcal M proteins^33^. Recently, a number of cross-reactive antibodies recognizing α-helical coiled-coil peptides derived from the orthologous sexual stage antigens of *P. falciparum* and *P. vivax* have been reported^34^. Peptides containing coiled-coil motifs from diverse *P. vivax* proteins could induce interferon γ production in immunized mice, suggesting the presence of T cell epitopes in the peptides^35^. While fine B-cell epitope mapping in PvMSP3γ awaits further investigation, it may not be entirely excluded that abundant coiled-coil structure spanning from variable blocks I to III occupying approximately 70% to 80% of this protein could elicit cross-reactive conformational B-cell epitopes between antigens CT1230N and CT1230C^12^ (Supplemental Fig. S5).

An ideal vaccine is expected to confer life-long immunity or, at least, a considerable period for protection against infection. Although it is conceivable that efficient immunity against malaria has to target antigens from multiple developmental stages of parasites that evoke complex interplay of host immune responses, complete protection from subsequent infections seems not to be generally achieved. The association between previous malaria episodes and anti-PvMSP3γ antibodies in terms of prevalence and magnitude of antibody reactivity has suggested that a vaccine derived from this protein could be boosted by natural malaria exposure. Pre-existing antibodies induced by natural infection have been proposed to be implicated in the low efficacy of *P. falciparum* circumsporozoite protein (CSP)-derived vaccines among volunteers living in malaria endemic areas, probably by competition or masking of protective epitopes in the vaccine constructs and interference with the activation of memory B cells^36^. However, several lines of evidence have suggested that naturally acquired antibodies to merozoite surface proteins could hinder the process of erythrocyte invasion by malarial merozoites^3^. PfMSP3 is one of the merozoite surface proteins that mediate immunity against *P. falciparum* infections among people in hyperendemic areas of Africa who have reached a state of premunition^4,6^. A subpopulation of anti-PfMSP3 antibodies is allegedly involved in ADCI, a process in which the antibodies cooperate with blood monocytes leading to parasite killing^4,37^. Nevertheless, the roles of PvMSP3 in inducing ADCI await further elucidation.

Unlike antibody-dependent cellular cytotoxicity (ADCC), ADCI recognizes FcγRII receptor on monocytes and is mediated by cytophilic subclasses of IgG antibodies (IgG1 and IgG3)^37^. A longitudinal cohort study in Ghanaian children has shown that ADCI is associated with reduced risk of symptomatic malaria^38^. Furthermore, vaccination trials in humans with hybrid proteins from glutamate-rich protein (GLURP) and PfMSP3 have shown that IgG3 against the latter immunogen plays a crucial role in ADCI^39^. In this study, anti-PvMSP3γ antibodies reactive to both variants were predominantly of the IgG1 and IgG3 subclasses with significantly higher reactivity index values than those of the IgG2 and IgG4 subclasses. A bias towards cytophilic antibodies has been reported in naturally acquired antibodies to PvMSP3α antigens^14,15^. Furthermore, a predominance of cytophilic antibody response has been observed in antibodies against other merozoite surface proteins of *P. vivax* that are vaccine targets^6,14,21,40^.

In this study, we did not include the Salvador I type antigen for analysis while there remain some possibilities that B epitopes spanning continuous regions of inserts A and C could differ from those in the Belem type antigen. Therefore, further fine epitope mapping on PvMSP3γ should include all three major types. Meanwhile, it could be possible to delineate whether antibody response direct against distinct or cross-reactive epitopes in this protein by competitive ELISA assay in future studies.

In conclusion, PvMSP3γ is immunogenic upon *P. vivax* infections although the rates of seropositivity in the same cohort seem to be lower than those directed against PvMSP9 and PvTRAP antigens. Based on analysis of antibody responses to recombinant PvMSP3γ antigens derived from two variants and subfragment domains, it is likely that B-cell epitopes were located across this protein. Anti-PvMSP3γ antibodies could be boosted by natural infections while the magnitude of antibody reactivity seemed to be positively associated with previous malaria exposure. Like PvMSP3α, IgG1 and IgG3 constituted the predominant IgG subclasses for anti-PvMSP3γ antibodies. Whether these cytophilic antibodies to PvMSP3γ could confer protection require further studies.

## Materials and methods

### Study population

Venous blood samples were collected from 246 *P. vivax*-infected patients at malaria clinics or district hospitals in Tak (n = 50) and Ubon Ratchathani Provinces (n = 196) during 2013 and 2014. After isolation of serum samples, they were aliquoted into cryotubes and stored at − 40 °C. These blood samples were previously analyzed for IgG antibody reactivity to recombinant PvTRAP and PvMSP9 antigens^20,21^. Negative control blood samples were from 50 healthy persons without known history of malaria exposure.

### Production of PvMSP3γ recombinant proteins

Our previous analysis of the *PvMSP3γ* complete coding sequences from 118 *P. vivax* isolates sampling from 4 major endemic areas of Thailandhas identified 86 distinct haplotypes^12^. Based on domain organization of this locus, the Belemtype was most common (74.6%), followed by the NR520 (22.0%) and Salvador1 (3.4%) types, respectively. Isolates CT1230 and NR25 were used as representative haplotypes for production of recombinant proteins because they were the predominant haplotypes belonging to the Belemand NR520 types, respectively^12^. Near complete coding sequences excluding the N-terminal 25 codons encoding the putative signal peptide of *PvMSP3γ* from isolates CT1230 and NR25 (corresponding to GenBank accession nos. MT363186 and MT363167) were included in recombinant protein production, designated CT1230nF and NR25nF, respectively (Fig. 1A). Two additional proteins derived from the N-terminal and C-terminal domains of isolate CT1230, designated CT1230N and CT1230C, were appended for subsequent comparison of antibody recognition. The CT1230N and CT1230C peptides shared 5 overlapping amino acids in the insert A domain (Fig. 1A). The near complete coding region of *PvMSP3γ* from isolate CT1230 was amplified by polymerase chain reaction (PCR) using primers PvCT1230nF-F (5′-CCCC**CTCGAG**AACGAAAATGTTAATTTGAAA-3′, *Xho*I site underlined) and PvCT1230nF-R (5′-CCCC**GGATCC**TTATTTAATTTTAAAAAGTGCATTCAC-3′, *Bam*HI site underlined). Primers PvNR25nF-F (5′-CCCC**CTCGAG**AACGAAAATGTGAATTTGAAAAATCCC-3′, *Xho*I site underlined) and PvNR25nF-R (5′-CCCC**GGATCC**TTATTTATTTTTAAAAGTTGCATTCACATC-3′, *Bam*HI site underlined) were used for amplification of the corresponding region of isolate NR25. PCR amplification of the subfragment CT1230N was performed by using forward primer PvCT1230N-F (5′-CCCC**CTCGAG**TCGGATGAAGCCTCACTC-3′, *Xho*I site underlined) and reverse primer PvCT1230N-C (5′-CCCC**GGATCC**TTATGCCTTACTTGATGTTTGT-3′, *Bam*HI site underlined) whose sequences were respectively derived from the 5′ region of variable block I and the 3′ end of the gene, respectively. For the subfragment CT1230C, forward primer PvCT1230C-F (5′CCCC**CTCGAG**CCATCAAGTAAGGCAGCAAAT-3′, *Xho*I site underlined) corresponding to the 3′ portion of insert block A and reverse primer PvCT1230C-R (CCCC**GGATCC**TTAACTTCCACTGGGGGATAC-3′, *Bam*HI site underlined) derived from conserved block III were used for PCR amplification (Fig. 1A). Amplification was performed in a total volume of 50 µL containing genomic DNA, 200 μM each deoxynucleotide triphosphate, 10 µL of 5× Phusion HF buffer, 0.5 µM each primer, and 1 unit of Phusion DNA Polymerase (New England Biolabs, MA, USA). The cycling condition for amplification contained an initial denaturation at 98 °C 30 s, followed by 35 cycles of 98 °C 10 s, 50 °C 30 s and 72 °C 80 s, and a final extension at 72 °C 5 min. The thermocycling conditions were essentially the same for all PCR targets while the reaction components were the same except that the corresponding forward and reverse primers were used. Analysis of the PCR products were performed by 1% agarose gel electrophoresis and purified by QIAquick Gel Extraction Kit (Qiagen, Hilden, Germany). Complete digestions of the purified PCR products with *Xho*I and *Bam*HI (New England Biolabs, MA, USA) were done prior to ligation into pET19b vector. *Escherichia coli* strain JM109 was used as a host for transformation. Verification of the recombinant plasmid DNA was performed by direct sequencing, followed by transformation into *E. coli* BL21 Star (DE3) pLysS expression host (Invitrogen, Carlsbad, CA). The procedures for protein expression comprising induction with isopropyl*-β*-d thiogalactopyranoside (IPTG), solubilization by sonication, refolding by multistep dialysis and purification using His-Bind Resin (Novagen, Germany) were done as previously described^20,21^.

### Enzyme-linked immunosorbent assay (ELISA).

Specific IgG antibodies to antigens CT1230nF, Ct1230N, CT1230C and NR25nF were determined by ELISA. High binding Costar ELISA plates were coated with 0.5 µg of the affinity purified antigen per well. The assays were done in duplicate wells using 100 μL of 1:100 dilution of individual plasma samples. Details on the procedures were essentially as described previously^20,21^. The mean optical density (OD_490_) values plus 3 standard deviations (S.D.) of 50 negative control serum samples were determined for each antigen and used as the cut-off values for positive titres. Human IgG subclasses were determined by ELISA following the procedures previously reported^21^. Biotin-conjugated isotype-specific mouse anti-human IgG subclasses (Sigma-Aldrich, Germany) with 1:1000 dilution were used as secondary antibodies. The cut-off values of antibody titres for recombinant antigens were derived from the mean OD_490_ values of negative control sera plus 3 S.D.

### Data analysis

The relative level of antibody reactivity to different antigens was estimated as the reactivity index (RI) by dividing the mean OD_490_ value of tested serum samples by the cut-off value for respective antigens. To compare differences between the numbers of responders to a given antigen, either Chi-square test or Fisher’s exact test was deployed. Mean amino acid distance value (*d*) measured the average evolutionary divergence of proteins based on pairwise comparison of amino acid sequences. Amino acid differences were calculated by using equal input model taken into account variation in frequencies among different kind of amino acids as follows:^41^

$$d\; = \; - b{\text{log }}\left( {{1 } - p/b} \right)$$

where *p* is the proportion of different amino acid sites, *g*_*i*_ is the frequency of amino acid *i*, and $b \; = \; {1 } - {\sum}_{{\text{i}}} g_{{\text{i}}}^{{2}}$.

The difference was assessed by z test. The nonparametric Spearman correlation coefficient was computed to determine the association between antibody reactivity between antigens. Interpretation of a correlation coefficient was based on absolute magnitude of the observed correlation coefficient levels taken into account the 95% confidence interval^24^. The nonparametric chi-square test for trend analysis and the Kruskal–Wallis test were used to determine the associations between antibody responses and prior malaria episodes. Differences between median RI values between pairs of IgG subclasses were analyzed by Mann–Whitney *U* test. Box plots representing antibody reactivity indices and Venn diagrams were illustrated by using Paleontological Statistics version 4.13 (PAST)^42^. Recombinant protein secondary structure was predicted and drawn by using The Structure Assessment service implemented in the SWISS-MODEL Workspace/GMQE^43^. Low complexity prediction of protein sequences was performed by using SEG^44^ and CAST^45^ algorithms implemented in the PlaToLoCo web meta-server^46^.

### Ethical approval

This study was reviewed and approved by the Institutional Review Board in Human Research of Faculty of Medicine, Chulalongkorn University, Thailand (IRB No. 546/58 and COA No. 041/2016). Prior to blood sample collection, informed consent was obtained from all participants or from their parents or guardians. All procedures were performed in accordance to the relevant guidelines and regulations.

### Supplementary Information

Supplementary Information.

## Data Availability

The datasets generated during and/or analyses during the current study are available from the corresponding author upon request.
