# Supplementary material for: Anti-Plasmodium vivax merozoite surface protein 3 ϒ (PvMSP3 ϒ) antibodies upon natural infection
Source: Sci Rep. 2024 Apr 26;14:9595. doi: 10.1038/s41598-024-59153-w (PMC11053162; doi:10.1038/s41598-024-59153-w)

Anti-*Plasmodium vivax* merozoite surface protein 3 gamma (PvMSP3 gamma) antibodies upon natural infection

Napaporn Kuamsab[1,2,3], Chaturong Putaporntip[1,*], Azumi Kakino[2], Rattiporn Kosuwin[1,2,4], Sunisa Songsaigath[1,2,4], Hiroshi Tachibana[2], Somchai Jongwutiwes[1,*]

[1]Molecular Biology of Malaria and Opportunistic Parasites Research Unit, Department of Parasitology, Faculty of Medicine, Chulalongkorn University, Bangkok, Thailand. [2]Department of Infectious Diseases, Tokai University School of Medicine, Isehara, Kanagawa, Japan; [3]Community Public Health Program, Faculty of Health Science and Technology, Southern College of Technology, Nakorn Si Thammarat, Thailand
[4]Department of Health Promotion, Faculty of Physical Therapy, Srinakharinwirot University, Nakhonnayok, Thailand

Supplementary Information

Contents

Supplemental Table S1. Rate of PvMSP3γ IgG seropositivity and gender of the patients…….2

Supplemental Table S2. Rate of PvMSP3γ IgG seropositivity and age group of the patients…3

Supplemental Table S3. Reactivity indices of PvMSP3γ IgG antibodies and age group of the patients……………………………………………………………………….…………………..….4

Supplemental Table S4. Profile of antibody reactivity to antigens CT1230nF, CT1230N and CT1230C…..………………………………………………………………………………..…5

Supplemental Table S5. PvMSP3γ IgG subclass antibodies among seropositive patients…….6

Supplemental Table S6 Previous malaria exposure status and number of antigens (PvMSP3γ, PvMSP9 and PvTRAP) recognized by IgG antibodies……………………………………….7

Supplemental Figure S1. Amino acid sequences of antigens NR25nF and CT1230nF relative to block boundaries of PvMSP3γ…………………………………………….…..….8

Supplemental Figure S2. Amino acid sequences of antigens CT1230nF, CT1230N and CT1230C relative to block boundaries of PvMSP3γ…………………………….…..……9

Supplemental Figure S3. Original SDS-PAGE image shown in the main article…….……..10

Supplemental Figure S4. Low complexity regions in the PvMSP3γ and PvMSP3α proteins predicted by SEG and CAST algorithms……………………………………………………11

Supplemental Figure S5. Tertiary structure of recombinant PvMSP3γ proteins predicted by using the SWISS-MODEL workspace…………………………………………..…….12

Supplemental Table S1. Rate of PvMSP3γ IgG seropositivity and gender of the patients

Gender	n	Antigen				
		**CT1230nF**	**CT1230N**	**CT1230C**	**NR25nF**	**Total#**
**No. positives antibodies to antigens (% responders for each gender)**						
Male	204	140 (68.63)	86 (42.16)	106 (51.96)	99 (48.53)	145 (71.08)
Female	42	30 (71.43)	22 (52.38)	20 (47.62)	21 (50.00)	31 (73.81)
p value*		0.125	0.057	0.737	0.313	0.851
**Mean reactivity indices ± S.E.**						
Male	204	0.883 ± 0.056	0.351 ± 0.032	0.871 ± 0.057	0.488 ± 0.043	
Female	42	1.128 ± 0.147	0.573 ± 0.130	0.947 ± 0.142	0.675 ± 0.137	
Two-sample paired *t* test = -2.6449, *p* value = 0.0773						

Positives for at least one antigen.

*Fisher’s exact test.

S.E. denotes standard error.

Supplemental Table S2 Rate of PvMSP3γ IgG seropositivity and age group of the patients

Age range (years)	n	No. reactive antibodies to antigen (% responders for age range)				
		CT1230nF	CT1230N	CT1230C	NR25nF	Total#
< 20	43	28 (65.12)	19 (44.19)	18 (41.86)	14 (32.56)	28 (65.12)
20 - 29	81	54 (66.67)	33 (40.74)	42 (51.85)	40 (49.38)	58 (71.61)
30 - 39	48	39 (81.25)	23 (47.92)	30 (62.50)	24 (50.00)	39 (81.25)
40 - 49	49	35 (71.43)	24 (48.98)	27 (55.10)	30 (61.22)	36 (73.47)
> 50	25	14 (56.00)	9 (36.00)	9 (36.00)	12 (48.00)	15 (60.00)
Chi-square for trend		0.001	0.008	0.035	3.839	0.005
p value		0.9993	0.9300	0.8517	0.0501	0.941

Positives for at least one antigen.

Supplemental Table S3 Reactivity indices of PvMSP3γ IgG antibodies and age group of the patients

Age range (years)	n	Median reactivity indices (interquartile range)			
		CT1230nF	CT1230N	CT1230C	NR25nF
< 20	43	1.560 (0.222, 2.280)	0.637 (0.148, 1.380)	0.787 (0.370, 2.583)	0.637 (0.223, 1.668)
20 - 29	81	1.525 (0.440, 3.497)	0.755 (0.310, 2.232)	1.132 (0.521, 3.210)	0.934 (0.443, 2.432)
30 - 39	48	1.888 (1.182, 2.979)	0.958 (0.255, 1.960)	1.641 (0.778, 3.747)	0.963 (0.459, 2.428)
40 - 49	49	1.583 (0.583, 3.485)	0.975 (0.430, 2.230)	1.422 (0.410, 3.276)	1.438 (0.354)
> 50	25	1.433 (0.274, 2.688)	0.506 (0.175, 2.281)	0.648 (0.340, 2.433)	0.895 (0.264, 2.133)
Kruskal-Wallis *H*		3.894	4.586	9.627	9.147
p value		0.4206	0.3324	0.0472#	0.0575

Exclusion of either age group < 20 or > 50 gave nonsignificant *p* values (H = 6.655, p = 0.0837 and H = 5.818, p = 0.1208).

Supplemental Table S4 Profile of antibody reactivity to antigens CT1230nF, CT1230N and CT1230C

Profile of antibody response to antigens			n	%
CT1230nF	CT1230N	CT1230C		
+	+	+	77	31.3
+	+	-	31	12.6
+	-	+	49	19.9
+	-	-	13	5.3
-	+	+	0	0
-	+	-	0	0
-	-	+	0	0
-	-	-	76	30.9
	Total		246	100

Supplemental Table S5. PvMSP3γ IgG subclass antibodies among seropositive patients

IgG subclass response	Antigen		
	CT1230N	CT1230C	NR25F
IgG1			
Ranges of OD values	0.052 - 1.084	0.060 - 1.288	0.063 - 3.217
Mean OD ± S.D.	0.265 ± 0.162	0.280 ± 0.180	0.512 ± 0.466
Median OD (IQR)	0.244 (0.167, 0.312)	0.262 (0.166, 0.356)	0.328 (0.246, 0.619)
Cut-off OD positive values	0.163	0.228	0.225
Frequency of responders (%)	83/108 (77.57)	95/126 (75.40)	98/120 (81.67)
IgG2			
Ranges of OD values	0.023 - 0.391	0.010 - 0.373	0.029 - 0.889
Mean OD ± S.D.	0.112 ± 0.065	0.076 ± 0.052	0.188 ± 0.148
Median OD (IQR)	0.109 (0.065, 0.137)	0.064 (0.041, 0.097)	0.135 (0.085, 0.241)
Cut-off OD positive values	0.130	0.101	0.195
Frequency of responders (%)	33/108 (30.84)	27/126 (21.43)	39/120 (32.50)
IgG3			
Ranges of OD values	0.070 - 1.892	0.053 - 1.126	0.067 - 1.460
Mean OD ± S.D.	0.294 ± 0.219	0.327 ± 0.145	0.365 ± 0.262
Median OD (IQR)	0.280 (0.198, 0.325)	0.342 (0.222, 0.393)	0.307 (0.217, 0.435)
Cut-off OD positive values	0.251	0.296	0.210
Frequency of responders (%)	68/108 (63.55)	92/126 (73.02)	93/120 (77.50)
IgG4			
Ranges of OD values	0.008 - 0.464	0.001 - 0.970	0.002 – 2.564
Mean OD ± S.D.	0.137 ± 0.079	0.104 ± 0.117	0.215 ± 0.288
Median OD (IQR)	0.307 (0.217, 0.435)	0.072 (0.035, 0.135)	0.153 (0.120, 0.196)
Cut-off OD positive values	0.191	0.118	0.263
Frequency of responders (%)	18/108 (16.82)	31/126 (24.60)	22/120 (18.33)

IQR, interquartile range

Supplemental Table S6 Previous malaria exposure status and number of antigens (PvMSP3γ, PvMSP9 and PvTRAP) recognized by IgG antibodies.

Previous malaria episode	Number of antigens reactive to antibodies		χ^2 with Yates correction (*p* value)
	1	> 1	
None (n = 91)	9	79	0.523
≥ 1 (n = 83)	5	78	(0.470)

Note: Of 174 patients with known previous malaria exposure, seronegatives to all antigens were found in 3 patients who did not have prior malaria exposure.

Supplemental Figure S1 Amino acid sequences of antigens NR25nF and CT1230nF relative to block boundaries of PvMSP3γ. The sequences of isolates CT1230 and NR25 are after GenBank accession nos. MT363186 and MT363167 (Reference 12). Asterisk and dash denote identical amino acid and deletion, respectively.

```
              ← Conserved block I                                                                →← Variable block I
MT363186      MKHLSGIPLL VLFLNLYVLQ NNVVSNENVN LKNPNLRNGW AGINLTQQDE QNGLGAEDGE EVNTKEDQNG LLHESDEASL QTQGQNS--- ---NEVAESN TLKKAKAAKD NAVKAEEDAE  [114]
CT1230nF                                   ***** ********** ********** ********** ********** ********** *******--- ---******* ********** **********  [89]
MT363167      ***FA***** ********** ********** ********** **K*V*L*** KIV*D**NRN Q*DSNGSHS* *PQKLEK*E* *****EKKKI NELDD**KQK QIE**NK**E G*L*QQKE**  [120]
NR25nF                                     ***** ********** **K*V*L*** KIV*D**NRN Q*DSNGSHS* *PQKLEK*E* *****EKKKI NELDD**KQK QIE**NK**E G*L*QQKE**  [95]

MT363186      KAKQKILEAV DKVKKAEEAI KNAVNKAKEV ADKAKEITTE KAKIAKEKAE ETAKLGKSVS ASFHALIAEK AETEAKEANE KAQMAAELAT HVANAYEAKK EAEKAQEVAE AAKREIEKLS  [234]
CT1230nF      ********** ********** ********** ********** ********** ********** ********** ********** ********** ********** ********** **********  [209]
MT363167      AQV*N*ED** K**E***D** *********A *N*****A** ***L*E***K K**EVKTYSV *K***GN*VT ***D****** **KK***T*K **V******* ******KL** **NKK**N**  [240]
NR25nF        AQV*N*ED** K**E***D** *********A *N*****A** ***L*E***K K**EVKTYSV *K***GN*VT ***D****** **KK***T*K **V******* ******KL** **NKK**N**  [215]

                                                                                                                  →← Insert block A
MT363186      KIYKEEKADT EEKSATAAAE SASQSVEKAK GEVGKAKEAA LNAAKNLTDA VEKLEKASEE LLKDNYLRDT VNSLKEGATE EQKKAKKEEE KAKISEEVAK AEAASAEAQF AKIEAERANY  [354]
CT1230nF      ********** ********** ********** ********** ********** ********** ********** ********** ********** ********** ********** **********  [329]
MT363167      *V**K*---- ---NV*E**Q ***KAA*E** AAAEE**A** *KV*QHVK** T****E**TQ **EYYKQSA* I*N**GEKEA *NEAI*EKAK I*AEAAKT*A EA*K------ ----------  [337]
NR25nF        *V**K*---- ---NV*E**Q ***KAA*E** AAAEE**A** *KV*QHVK** T****E**TQ **EYYKQSA* I*N**GEKEA *NEAI*EKAK I*AEAAKT*A EA*K------ ----------  [312]

                                                                                                                  →← Insert block B
MT363186      EANKIAENHP NTNVTEEANK AKVASTKAST EATKATKAST EATKASTEAT NASTEATKPS SKAANVKKKT DEAIKAAKEA KKAKTEAYIA LFVTKAMAAK EKAKKSAEAA DKAKAQAEAV  [474]
CT1230nF      ********** ********** ********** ********** ********** ********** ********** ********** ********** ********** ********** **********  [449]
MT363167      ---------- ---------- ---------- ---------- ---------- ---------- ---------- ---------- ---------- ---------- ---------- ----------  [337]
NR25nF        ---------- ---------- ---------- ---------- ---------- ---------- ---------- ---------- ---------- ---------- ---------- ----------  [312]

                                                                                                             →← Insert block C
MT363186      NGASEKTKKD AEHAATKANE KKTHTETAAD AAKKNAEVKV EEEDNVAKNE EKMKKKVDDV IEKVLEALKS EEDTYQAQIQ AEIAVQVANV EVACEKAKTA EQEAKKAKDE AVKAAKEAEE  [544]
CT1230nF      ********** ********** ********** ********** ********** ********** ********** ********** ********** ********** ********** **********  [569]
MT363167      ---------- ---------- ---------- ---------- ---------- ---------- ---------- ---------- ---------- ---------- ---------- ----------  [337]
NR25nF        ---------- ---------- ---------- ---------- ---------- ---------- ---------- ---------- ---------- ---------- ---------- ----------  [312]

MT363186      AKKQAEKAEK ITKTATEEAN KAKEEEAKAS EAKQEAETKA GDVDEEVYAV NVEFESVKAA AKAAAHHKVP EILDKEKKNA ENSAKKASAN ANDATTIAAT ANTKATEAKN AADKAQKASE  [714]
CT1230nF      ********** ********** ********** ********** ********** ********** ********** ********** ********** ********** ********** **********  [689]
MT363167      ---------- ---------- ---------- ---------- ---------- ---------- ---------- ---------- ---------- ---------- -----*A*EA **EN***E**  [352]
NR25nF        ---------- ---------- ---------- ---------- ---------- ---------- ---------- ---------- ---------- ---------- -----*A*EA **EN***E**  [327]

                 →← Conserved block II                                  →← Variable block II                     →← Conserved block III
MT363186      NAKAIAADVL AQKASREAQS LKQEAEKLAE DIQKSNVTNE EKEKAQKAAK EAKDAAEQAS TSANKANEAK TATIGAEGEN ALQKKKEEST KAVNASKEAM KARDKAAFEL LKIKKQDVLE  [834]
CT1230nF      ********** ********** ********** ********** ********** ********** ********** ********** ********** ********** ********** **********  [809]
MT363167      KV*****Y** *****TQ**L **E******* N*K**K**D* **T**DE*** A*****G*** A*TKT**D** **ATK*D*GE T*E******A ********** ********** **********  [472]
NR25nF        KV*****Y** *****TQ**L **E******* N*K**K**D* **T**DE*** A*****G*** A*TKT**D** **ATK*D*GE T*E******A ********** ********** **********  [447]

                                                    →← Variable block III                  →← Conserved block IV
MT363186      QVDVSPSGSD NLNDVDEQVA LEVGEQQNET EDAEPQETEE GDE-EDEEDE EDEEETEEEE IQDENDHTEE SSAKQAAEQE KQQGEKVLND GEAHNLLAQQ HKKDKNAATE DAKLFQTIIK  [953]
CT1230nF      ********** ********** ********** ********** ***-****** ********** ********** ********** ********** ********** ********** **********  [928]
MT363167      P**I*SNDNI *I*******V *********S ***PS**GG* *T*VG*D**L *Y******** **EGDT*KK* A*T***V*K* **KS*E**** ED*L****KL *N**N*D*** N*********  [592]
NR25nF        P**I*SNDNI *I*******V *********S ***PS**GG* *T*VG*D**L *Y******** **EGDT*KK* A*T***V*K* **KS*E**** ED*L****KL *N**N*D*** N*********  [567]

                                     →
MT363186      NFEDDDDFKN LQTGVNALFK IK [975]
CT1230nF      ********** ********** ** [950]
MT363167      DVDGG***** ***D***T** N* [614]
NR25nF        DVDGG***** ***D***T** N* [589]
```

Supplemental Figure S2 Amino acid sequences of antigens CT1230nF, CT1230N and CT1230C relative to block boundaries of PvMSP3γ. Isolate CT1230 sequence is after GenBank accession no. MT363186 (Reference 12). Asterisk and dash denote identical amino acid and deletion, respectively.

```
            ← Conserved block I                                                           →← Variable block I
MT363186    MKHLSGIPLL VLFLNLYVLQ NNVVSNENVN LKNPNLRNGW AGINLTQQDE QNGLGAEDGE EVNTKEDQNG LLHESDEASL QTQGQNSNEV AESNTLKKAK AAKDNAVKAE EDAEKAKQKI  [120]
CT1230nF                                   ***** ********** ********** ********** ********** ********** ********** ********** ********** **********  [95]
CT1230N                                                                                   ****** ********** ********** ********** **********  [95]

MT363186    LEAVDKVKKA EEAIKNAVNK AKEVADKAKE ITTEKAKIAK EKAEETAKLG KSVSASFHAL IAEKAETEAK EANEKAQMAA ELATHVANAY EAKKEAEKAQ EVAEAAKREI EKLSKIYKEE  [240]
CT1230nF    ********** ********** ********** ********** ********** ********** ********** ********** ********** ********** ********** **********  [215]
CT1230N     ********** ********** ********** ********** ********** ********** ********** ********** ********** ********** ********** **********  [215]

                                                                                                               →← Insert block A
MT363186    KADTEEKSAT AAAESASQSV EKAKGEVGKA KEAALNAAKN LTDAVEKLEK ASEELLKDNY LRDTVNSLKE GATEEQKKAK KEEEKAKISE EVAKAEAASA EAQFAKIEAE RANYEANKIA  [360]
CT1230nF    ********** ********** ********** ********** ********** ********** ********** ********** ********** ********** ********** **********  [335]
CT1230N     ********** ********** ********** ********** ********** ********** ********** ********** ********** ********** ********** **********  [335]

                                                                                                               →← Insert block B
MT363186    ENHPNTNVTE EANKAKVAST KASTEATKAT KASTEATKAS TEATNASTEA TKPSSKAANV KKKTDEAIKA AKEAKKAKTE AYIALFVTKA MAAKEKAKKS AEAADKAKAQ AEAVNGASEK  [480]
CT1230nF    ********** ********** ********** ********** ********** ********** ********** ********** ********** ********** ********** **********  [455]
CT1230N     ********** ********** ********** ********** ********** *******                                                                     [392]
CT1230C                                                              ******** ********** ********** ********** ********** ********** **********  [68]

                                                                                                        →← Insert block C
MT363186    TKKDAEHAAT KANEKKTHTE TAADAAKKNA EVKVEEEDNV AKNEEKMKKK VDDVIEKVLE ALKSEEDTYQ AQIQAEIAVQ VANVEVACEK AKTAEQEAKK AKDEAVKAAK EAEEAKKQAE  [600]
CT1230nF    ********** ********** ********** ********** ********** ********** ********** ********** ********** ********** ********** **********  [600]
CT1230C     ********** ********** ********** ********** ********** ********** ********** ********** ********** ********** ********** **********  [188]

                                                                                                                                     →←
MT363186    KAEKITKTAT EEANKAKEEE AKASEAKQEA ETKAGDVDEE VYAVNVEFES VKAAAKAAAH HKVPEILDKE KKNAENSAKK ASANANDATT IAATANTKAT EAKNAADKAQ KASENAKAIA  [720]
CT1230nF    ********** ********** ********** ********** ********** ********** ********** ********** ********** ********** ********** **********  [720]
CT1230C     ********** ********** ********** ********** ********** ********** ********** ********** ********** ********** ********** **********  [308]

            Conserved block II                           →← Variable block II                   →← Conserved block III
MT363186    ADVLAQKASR EAQSLKQEAE KLAEDIQKSN VTNEEKEKAQ KAAKEAKDAA EQASTSANKA NEAKTATIGA EGENALQKKK EESTKAVNAS KEAMKARDKA AFELLKIKKQ DVLEQVDVSP  [840]
CT1230nF    ********** ********** ********** ********** ********** ********** ********** ********** ********** ********** ********** **********  [840]
CT1230C     ********** ********** ********** ********** ********** ********** ********** ********** ********** ********** ********** **********  [428]

                                           →← Variable block III                   →← Conserved block IV
MT363186    SGSDNLNDVD EQVALEVGEQ QNETEDAEPQ ETEEGDEEDE EDEEDEEETE EEEIQDENDH TEESSAKQAA EQEKQQGEKV LNDGEAHNLL AQQHKKDKNA ATEDAKLFQT IIKNFEDDDD  [960]
CT1230nF    ********** ********** ********** ********** ********** ********** ********** ********** ********** ********** ********** **********  [960]
CT1230C     ***                                                                                                                                [431]

                           →
MT363186    FKNLQTGVNA LFKIK [975]
CT1230nF    ********** ***** [975]
```

Supplemental Figure S3. Original SDS-PAGE image shown in the main article.

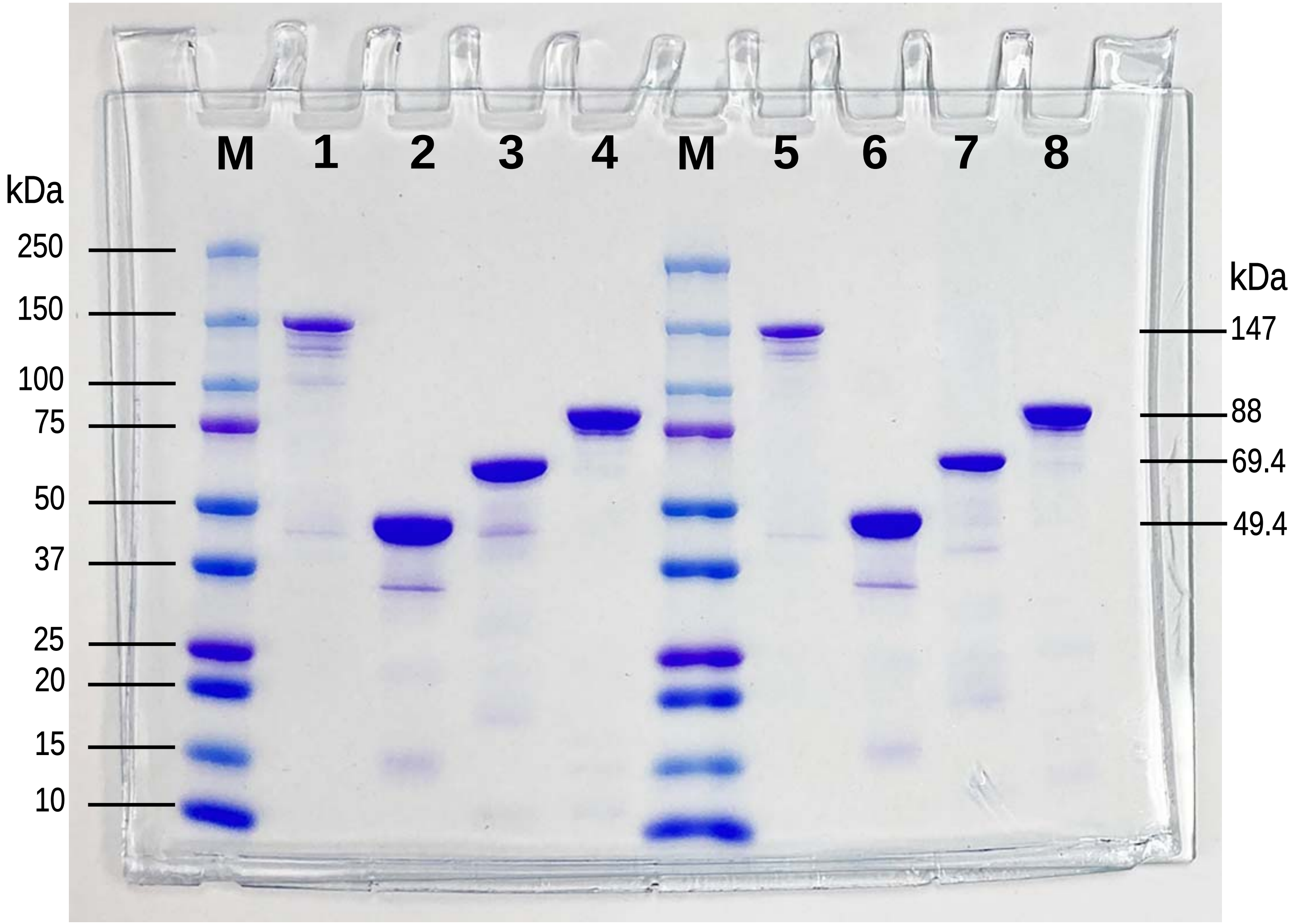

Supplemental Figure S4. Low complexity regions (blue boxes) in PvMSP3γ proteins predicted by SEG and CAST algorithms[43-45] Boxes underneath are after Fig. 1A

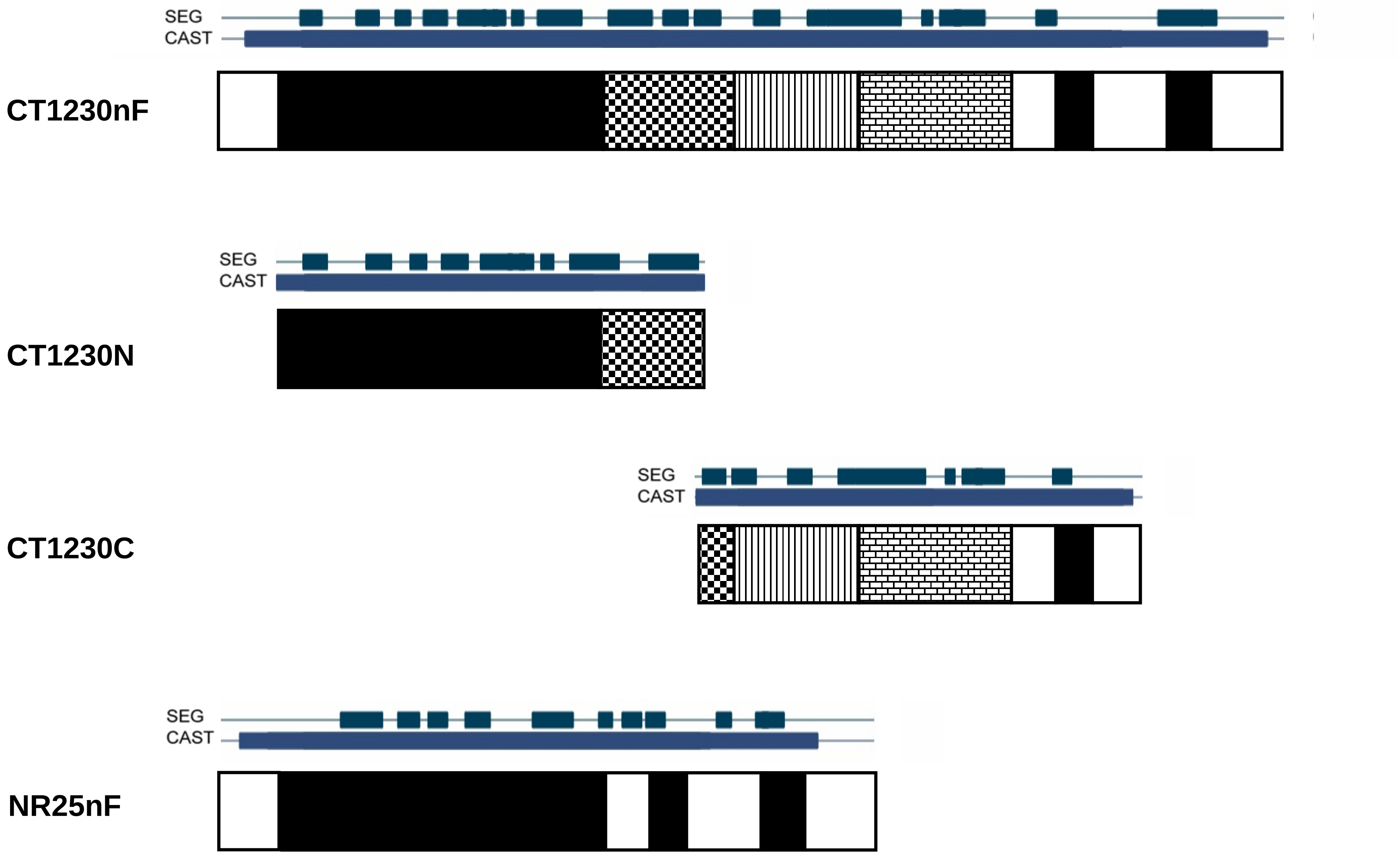

Supplemental Figure S5. Tertiary structure of recombinant PvMSP3γ proteins predicted by using the SWISS-MODEL workspace. A, C, E and G represent CT1230nF, CT1230N, CT1230C and NR25nF, respectively. B, D, F and H are horizontal 180° rotation of the corresponding proteins.

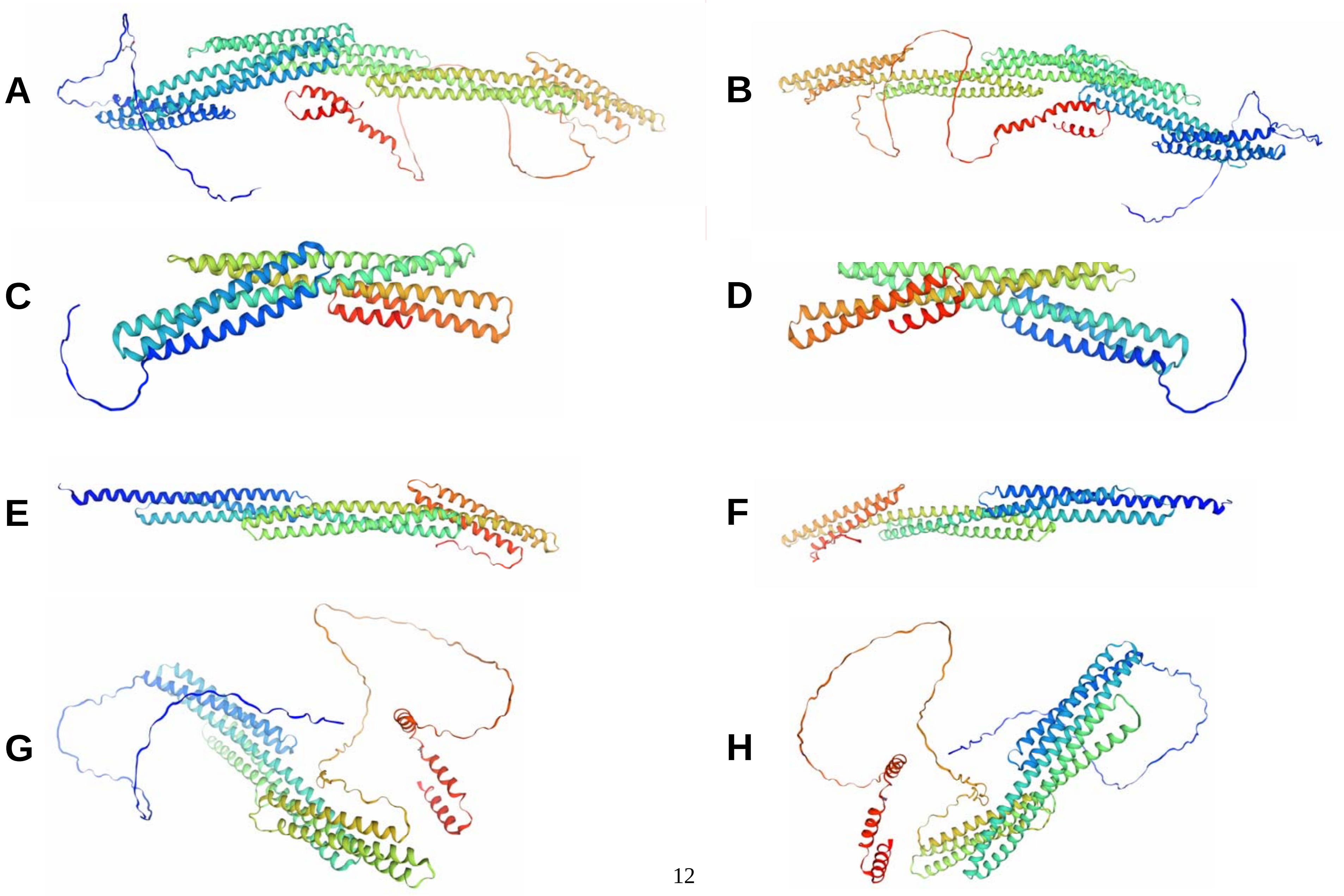

Supplement: Supplementary file 1 — Supplementary Information. [file 41598_2024_59153_MOESM1_ESM.pdf]
